# Machine learning methods for automated classification of tumors with papillary thyroid carcinoma-like nuclei: A quantitative analysis

**DOI:** 10.1371/journal.pone.0257635

**Published:** 2021-09-22

**Authors:** Moritz Böhland, Lars Tharun, Tim Scherr, Ralf Mikut, Veit Hagenmeyer, Lester D. R. Thompson, Sven Perner, Markus Reischl

**Affiliations:** 1 Institute for Automation and Applied Informatics, Karlsruhe Institute of Technology, Eggenstein-Leopoldshafen, Germany; 2 Institute of Pathology, University of Luebeck and University Hospital Schleswig-Holstein Campus Luebeck, Luebeck, Germany; 3 Department of Pathology, Woodland Hills Medical Center, Southern California Permanente Medical Group, Woodland Hills, Los Angeles, California, United States of America; Kameda Medical Center, JAPAN

## Abstract

When approaching thyroid gland tumor classification, the differentiation between samples with and without “papillary thyroid carcinoma-like” nuclei is a daunting task with high inter-observer variability among pathologists. Thus, there is increasing interest in the use of machine learning approaches to provide pathologists real-time decision support. In this paper, we optimize and quantitatively compare two automated machine learning methods for thyroid gland tumor classification on two datasets to assist pathologists in decision-making regarding these methods and their parameters. The first method is a feature-based classification originating from common image processing and consists of cell nucleus segmentation, feature extraction, and subsequent thyroid gland tumor classification utilizing different classifiers. The second method is a deep learning-based classification which directly classifies the input images with a convolutional neural network without the need for cell nucleus segmentation. On the Tharun and Thompson dataset, the feature-based classification achieves an accuracy of 89.7% (Cohen’s Kappa 0.79), compared to the deep learning-based classification of 89.1% (Cohen’s Kappa 0.78). On the Nikiforov dataset, the feature-based classification achieves an accuracy of 83.5% (Cohen’s Kappa 0.46) compared to the deep learning-based classification 77.4% (Cohen’s Kappa 0.35). Thus, both automated thyroid tumor classification methods can reach the classification level of an expert pathologist. To our knowledge, this is the first study comparing feature-based and deep learning-based classification regarding their ability to classify samples with and without papillary thyroid carcinoma-like nuclei on two large-scale datasets.

## Introduction

The seminal challenge in thyroid gland pathology is to determine whether the magnitude of nuclear alterations present in a sample is sufficient to be considered “papillary thyroid carcinoma-like” (PTC-like). PTC-like nuclear alterations are the major discriminator between four of the most common benign and malignant thyroid gland neoplasms, all of which can show an encapsulated follicular-patterned tumor: noninvasive follicular thyroid neoplasm with papillary-like nuclear features (NIFTP) and follicular variant of papillary thyroid carcinoma (FVPTC), which both exhibit PTC-like nuclei, versus follicular adenoma (FA) and follicular thyroid carcinoma (FTC), both of which do not show PTC-like nuclei [1]. Accurate discrimination between NIFTP and FVPTC in particular poses a major challenge to pathologists. This discrimination requires incorporation of architectural features (e.g. papillae formation, invasion), which is beyond the scope of this nucleus-centered study. Since NIFTP are known to exhibit less clear PTC-like nuclei, we chose the Nikiforov box A cohort for analysis, as this was assembled to define NIFTP and is therefore enriched in cases with borderline nuclear features, difficult to classify even for expert pathologists. The utility of assisted nuclear feature assessment lies in being able to evaluate every single neoplastic nucleus in a standardized manner, thereby reducing interpretation errors due to tumor heterogeneity and individual thresholds. An automated designation of any area of PTC-like nuclei in a neoplasm could therefore potentially change the diagnosis from benign to malignant (i.e. FA to FVPTC) if papillae are also present. Vice versa, a neoplasm erroneously regarded as FVPTC by subjective designation of PTC-like nuclei might represent FA by standardized evaluation for PTC-nuclei. Additionally, application of automated nuclear evaluation to cytology specimen as well might prove valuable to improve intra- and interobserver consistency.

Pathologists utilize several morphological criteria to determine whether a tumor is considered PTC-like or not, hence non-PTC-like. These criteria predominantly include changes in nuclear size and shape, nuclear membrane irregularities, and chromatin distribution features. Since the criteria are qualitative, there is a well-recognized inter-observer variability among pathologists in the overall weighting of criteria for PTC-like nuclei [2, 3]. Additionally, the nuclear alterations are often heterogeneously and unevenly distributed in a single tumor nodule (so called “sprinkling sign”), requiring a meticulous and time-consuming evaluation. To reduce inter-observer variability and speed up the process of determining ultimate tumor classification, machine learning methods could provide an objective, thorough, and reproducible method to assist pathologists in making these distinctions.

In the past, several studies focused on automated classification of thyroid tumors. In [4], the deep learning network architectures Inception-ResNetV2 and VGG19 are compared in their capacity to classify normal thyroid tissue, adenoma, nodular goiter, PTC, FTC, medullary thyroid carcinoma (MTC), and anaplastic thyroid carcinoma (ATC). The cohort for their fully-automated method consisted of 806 patients and the images were acquired with a 100*x* magnification. The cohort was labeled by two senior pathologists; any discordant cases were removed, resulting in removal of potentially difficult or challenging cases.

Wei Wang, et al., worked on distinguishing normal thyroid tissue, FA, and FTC [5]. The cohort consisted of 10 samples: five FA and five FTC samples. Images were acquired using 400*x* magnification (0.074 μm/px) and 24bit RGB channels. In contrast to most studies using hematoxylin and eosin (H&E) staining, Feulgen staining was used. With Feulgen staining, only DNA is stained, resulting in a better visibility of the nuclei for computer recognition. The method consists of a semi-automated segmentation, reviewed by an expert pathologist, and followup feature extraction and classification. Solely the nuclear area or the entropy of intensity values was statistically significant enough to achieve 100% accuracy. Regarding the study size, the authors mention, that “10 human cases is not nearly enough to establish the validity of the technology in a clinical sense” [5].

In [6], normal thyroid tissue and PTC were classified. A segmentation pipeline using particle swarm optimization-based Otsu’s multilevel thresholding was used. Binary images were manually extracted and post-processed, resulting in a semi-automated approach. Gray level co-occurrence matrix features were created and a closest-matching-rule algorithm was proposed for classification. The cohort consisted of 12 patients and images were taken at 40*x* magnification. The ground-truth classification was performed by a pathologist.

In [7] a neural network able to predict the BRAF-RAS gene expression signature is used to identify NIFTP. The dataset was trained on a network extracted from The Cancer Genome Atlas and has 497 individual samples. Furthermore, an Xception-based network was used to classify papillary thyroid carcinoma classic, papillary thyroid carcinoma extensive follicular growth, NIFTP and FA. The dataset has 115 samples scanned with 40*x* magnification. The images used for training have a resolution of 1.01 μm/px with an effective optical magnification of 10*x*. The trained network is able to detect NIFTP samples with a sensitivity of 89.4% and a specificity of 89.7%, thus correlating morphology to RAS-like gene expression signature.

The authors of [8] used weakly supervised instance learning to predict the malignancy of thyroid tumor from whole slide images. The Bethesda System (TBS) diagnostic score is used as a classification target. The dataset contained 908 cases scanned with 40*x* magnification resulting in a resolution of 0.25 μm/px. An area under curve of 0.870 ± 0.017 with an average precision of 0.743 ± 0.037 was achieved.

The previous studies show that not only a multitude of different methods with various parameterizations can be applied, but also that datasets are heterogeneous and adapted to the needs of each lab. To apply machine learning methods to histopathology and assist pathologists in classification of a tumor, e.g., PTC-like vs. non-PTC-like, pathologists need to know what methods can be applied and what level of accuracy can be expected for their data.

While feature-based classification in [5] and deep learning-based classification in [4] have individually proven their applicability for thyroid tumor classification, there is no direct comparison of both methods nor is it known which method should be used and how it may need to be parameterized.

The present paper is addressed to pathologists with an interest in machine learning and bioinformaticians with an interest in histology. We start by introducing the two dominant approaches for thyroid tumor classification: feature-based and deep learning-based. This enables pathologists to decide which method is suitable for their data, while it enables bioinformaticians to apply and adapt the methods to their individual problem setting. Pathologists and bioinformaticians need to be enabled to make this selection, since thyroid tumor datasets are not standardized for histochemical staining nor image acquisition, and therefore high quality results on new data requires individual method selection and adaption. In our experiments, the methods are applied to two datasets. Further, for one dataset we compare the results for each sample to the results of 24 expert thyroid gland pathologists. We aim to assist pathologists in evaluating the methods for clinical use in terms of accuracy, interpretability, and computational complexity, and enable them to assess whether individual training of the methods for their datasets is necessary. Furthermore, we demonstrate that tumor classification feature-based techniques are comparable to deep learning-based techniques. Both datasets were created with standard imaging techniques and resolutions of 0.23 μm/px (40x magnification) and 0.49 μm/px (20x magnification). We show that this is sufficient to achieve high quality results. Finally, results are discussed and conclusions for practical application of automated thyroid tumor classification methods are shown.

## Methods

### Overview

In this section, two datasets and two supervised methods able to classify histopathological images of thyroid tumors into PTC-like and non-PTC-like are introduced. While the feature-based classification consists of a three stage process (segmentation, feature extraction and classification), the deep learning-based classification is directly performed on the thyroid images, coupled with the diagnosis. An implementation for both methods is available at https://github.com/moboehland/thyroidclassification. The Ethics Committee of the Universität zu Lübeck approved our study. The commission has no concerns regarding our study, gave written consent, and the internal reference number is 20–267. The performed study was retrospective and all data was fully anonymized. The ethics committee waived the requirement for informed consent.

### Datasets

**Fig 1:**
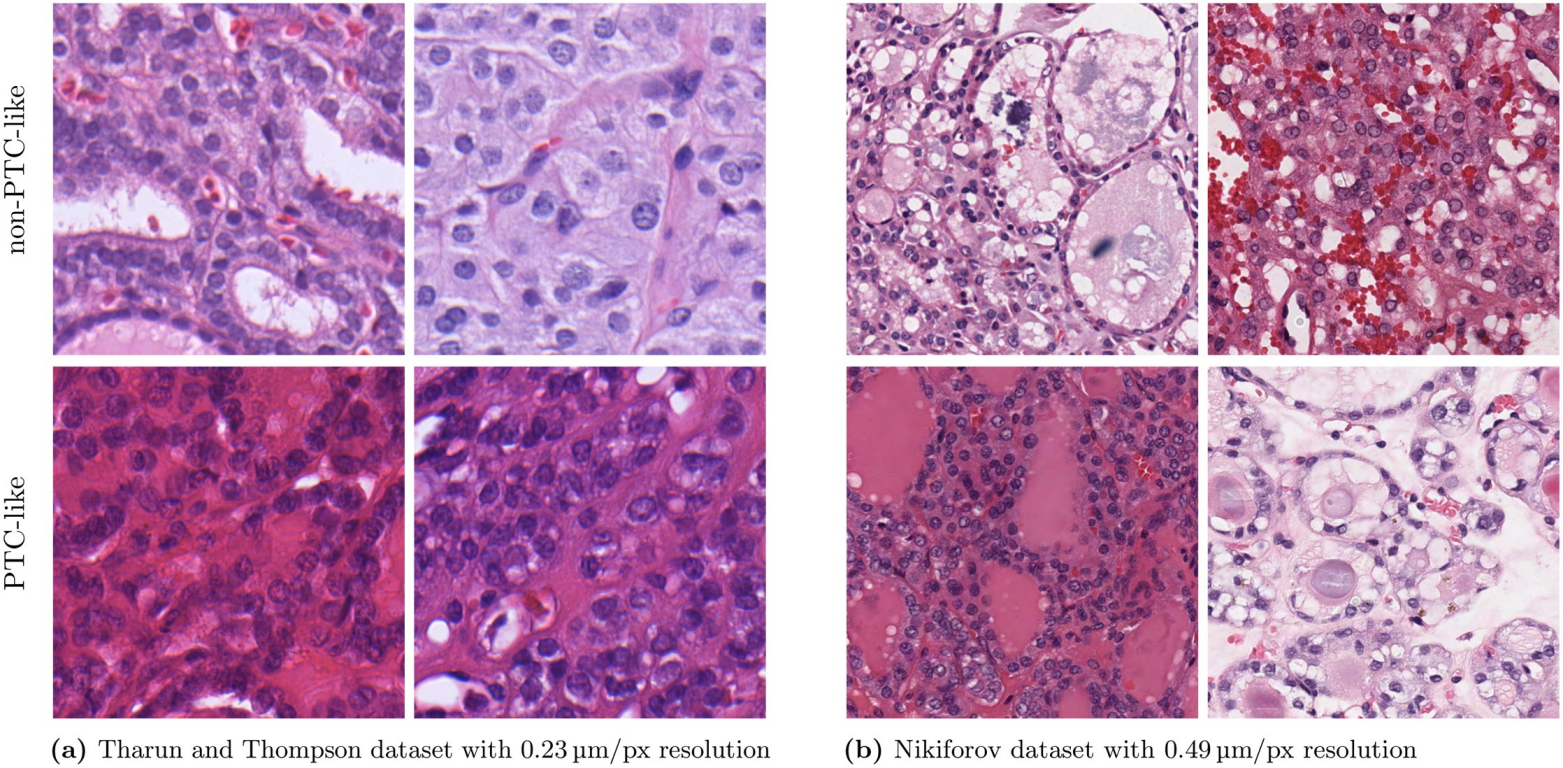
Example thyroid gland tumor images from the Tharun and Thompson dataset and the Nikiforov dataset. The images are cropped to 512 × 512 px. The upper row shows non-PTC-like samples and the lower row shows PTC-like samples. A high-resolution version is available in S1 Fig.

Since machine learning approaches and especially deep learning approaches heavily depend on the underlying data, two different datasets were used for the experiments. Histology examples of PTC-like and non-PTC-like images are shown in Fig 1.

#### Tharun and Thompson dataset

The Tharun and Thompson dataset consists of a cohort of 156 thyroid gland tumors built from the pathology archives at the University Clinic Schleswig-Holstein, Campus Luebeck (n = 138) and the Woodland Hills Medical Center, Woodland Hills, California (n = 18). A representative, hematoxylin and eosin-stained section per tumor was selected and scanned with the Ventana iScan HT (Roche diagnostics, Basel, Switzerland). Whole slide images were acquired at 40*x* magnification and a resolution of 0.23 μm/px. Whole slide images were processed as 8-bit color depth RGB images. Afterwards, two pathologists agreed upon the classification for each whole slide image. Divergent diagnoses were resolved by discussion, yielding consensus on every case. The dataset can be requested by sending an email to sekretariat.patho@uksh.de.

The dataset was divided into the following five entities:

- follicular thyroid carcinoma (FTC, 32 patients),
- follicular thyroid adenoma (FA, 53 patients),
- noninvasive follicular thyroid neoplasm with papillary-like nuclear features (NIFTP, 9 patients),
- follicular variant papillary thyroid carcinoma (FVPTC, 9 patients), and
- classical papillary thyroid carcinoma (PTC, 53 patients).

Oncocytic neoplasms were excluded as these are readily classifiable by their cytoplasmic and architectural features. For this study, the five different entities were combined into non-PTC-like (FTC, FA, 85 patients) and PTC-like (NIFTP, FVPTC, and PTC, 71 patients). For the experiments, a pathologist extracted representative images from neoplastic areas from each whole slide image. For 147 out of 156 entities, 10 images of size 1916 × 1053 px without overlap from neoplastic areas were extracted. For the remaining nine cases the neoplasm areas are small and only one to six images are available. The images were extracted with the acquisition magnification of 40*x* and a resolution of 0.23 μm/px.

#### Nikiforov dataset

The Nikiforov dataset consists of the two parts BoxA and BoxB and is available at http://image.upmc.edu:8080/NikiForovEFVStudy/view.apml. For classification into PTC-like and non-PTC-like tumors, Box A was used. Images were available at 20*x* magnification with a resolution of 0.49 μm/px. All 138 cases were initially submitted by six different institutions as potential encapsulated follicular variant papillary thyroid carcinoma (EFVPTC) to define NIFTP out of these.

Each case was afterwards individually classified by an international panel of 24 expert thyroid pathologists into one of the five entities:

- encapsulated follicular variant papillary thyroid carcinoma (EFVPTC, enriched in NIFTP according to the then defined nomenclature),
- invasive follicular variant papillary thyroid carcinoma (IFVPTC),
- classical papillary thyroid carcinoma (CPTC),
- follicular thyroid carcinoma (FTC), and
- benign (this category included follicular adenoma, goiter, nodular hyperplasia, adenomatoid nodules).

These five entities were grouped into entities with PTC-like nuclei (EFVPTC, IFVPTC, and CPTC) and non-PTC-like nuclei (FTC and benign). A list with the number of pathologists classifying each case into each entity was provided to us by the authors of [9], while the scoring of each individual pathologist was not available to us.

Furthermore, in the study’s supplementary material, 30 cases have been reviewed again by 23 pathologists, where a nuclear score of two or three defines PTC-like nuclei. We excluded two cases (A38 and A120) since only 13 and 15 pathologists defined them as PTC-like. All cases with less than 13 pathologists rating the case as PTC-like have been classified as non-PTC-like, while all cases with more than 15 pathologists rating the case as PTC-like have been classified as PTC-like. All other cases not reviewed again in the study’s supplementary material have been classified according to the majority vote in the list made available to us. Three additional cases have been removed since the same amount of pathologists rendered each case PTC-like and non-PTC-like. The final dataset consists of 103 PTC-like and 30 non-PTC-like diagnoses.

Due to the initially submitted classification as EFVPTC with the intention to find a cohort defining NIFTP, we conclude that the dataset contains many borderline cases that were difficult to classify even by expert pathologists and is therefore labeled as a more difficult dataset.

Since the anonymized classification by the thyroid expert pathologists is available to us, the results of the machine learning methods can be compared to the classifications of the expert pathologists. Additionally, it could be determined whether the samples difficult to classify by expert pathologists are also difficult to classify by machine learning methods.

The classification is available for each whole slide image. To be comparable to the Tharun and Thompson dataset, an image of size 2272 × 2272 px is extracted from neoplastic areas for each whole slide image by a pathologist. This results in approximately the same area in μm available for each case compared to the Tharun and Thompson dataset. The images were extracted with the acquisition magnification of 20*x* and a resolution of 0.49 μm/px. For the feature-based classification, images were upscaled to 40x (by a factor of two) using spline interpolation of order three. This was done to match the magnification of the Tharun and Thompson dataset and the Multi-Organ Nuclei Segmentation Challenge dataset [10, 11], which is used to train the segmentation model for the feature-based classification.

### Feature-based classification

**Fig 2:**
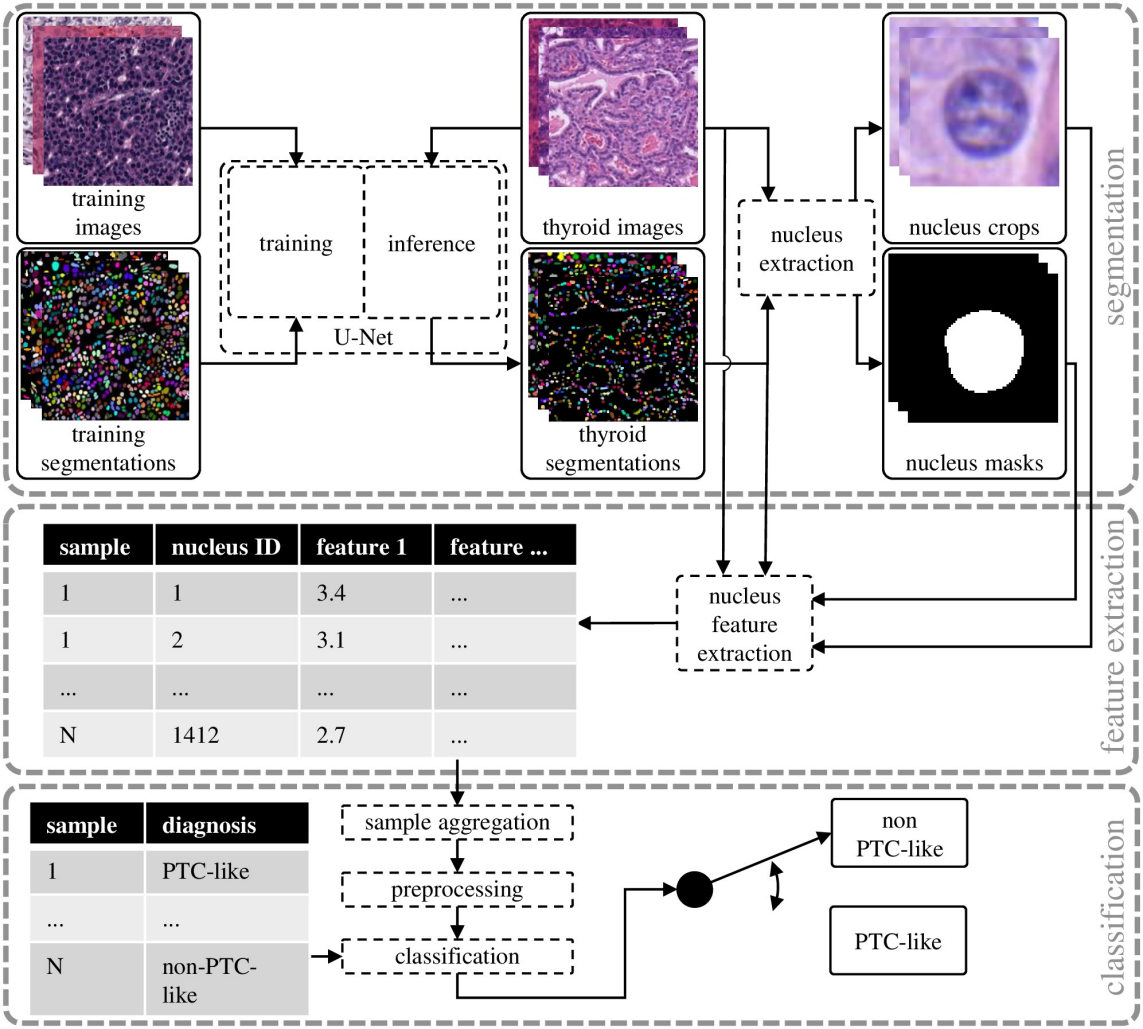
The feature-based classification consisting of segmentation, feature extraction and classification.

The first step of the feature-based classification shown in Fig 2 is the segmentation where the thyroid images are used to extract the thyroid segmentations, nucleus crops, and nucleus masks. Afterwards, during feature extraction, the thyroid images, the corresponding segmentations, and the extracted nucleus crops and nucleus masks are used to extract nucleus features. During the classification step, the extracted features are aggregated, preprocessed and used for classification with nested cross-validation. This method has been used in former studies on small datasets or images with high magnification (100*x* or more) with semi-automated cell nucleus segmentation approaches, where pathologists reviewed the segmentation [5, 6, 12].

#### Cell nucleus segmentation

**Fig 3:**
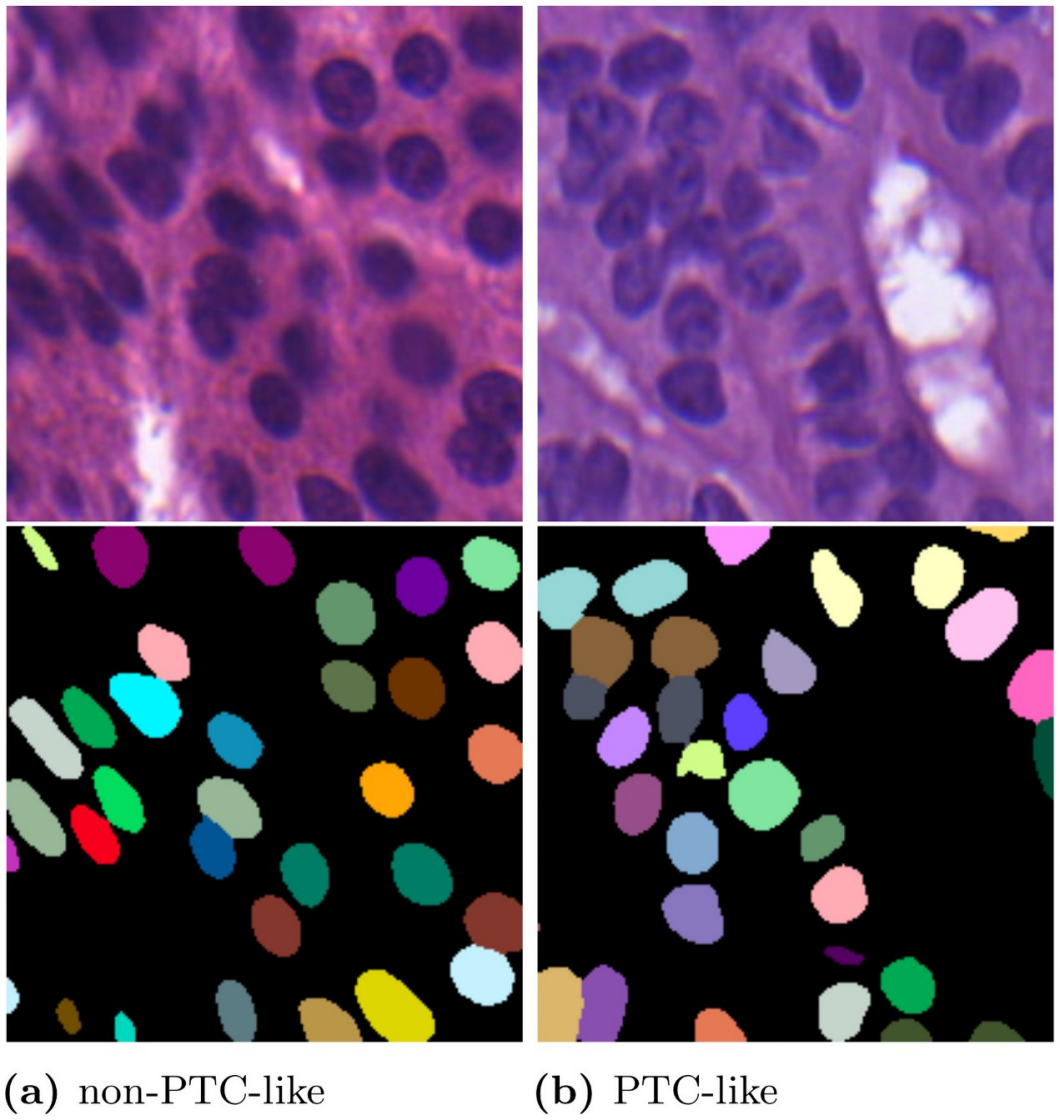
Exemplary thyroid images from the Tharun and Thompson dataset and the corresponding instance segmentations. The images are 200 × 200 px crops. The left images show a non-PTC-like sample and the right images a PTC-like sample.

The aim of the cell nucleus segmentation is to extract the shape and position of each individual nucleus in the image. This instance segmentation is often visualized color-coded, i.e., each detected nucleus gets an unique label which is assigned to a different color. Artifacts like nuclei not being fully visible in the image plane should not be segmented. Exemplary instance segmentations are shown in Fig 3.

In former studies, the cell segmentation step was performed with semi-automated methods without utilizing deep learning methods [5, 6]. One possibility to fully automate the cell segmentation is to use convolutional neural networks which can be used as there are a number of histopathology datasets available that provide cell nucleus annotations [10, 13]. Convolutional neural networks (i.e., the U-Net [14]), have proven their ability to generate high quality segmentation results [15]. The U-Net architecture is commonly used in biological cell segmentation tasks and provides state-of-the-art results [10, 15, 16]. For the segmentation, we use the double encoder U-Net and the adapted border method [16]. Deep learning is only used during the segmentation step in the feature-based classification. This adaption to the method is undertaken to increase segmentation quality and omit manual review of the segmentation.

To train the segmentation model, we use the training data provided by the Multi-Organ Nuclei Segmentation Challenge [10, 11], although it does not contain any thyroid images. Still, as shown in Fig 3 the training data is sufficient to enable inference on thyroid images. The main problems after inference on new thyroid images are merged nuclei and nuclei that are not sufficiently in the image plane. Not removing those segmentations would bias the features created. Both problems are addressed by a post-processing. All nuclei with an area larger than 200% of the median nucleus (merged nuclei) and smaller than 50% of the median nucleus (nuclei insufficiently represented in the image plane) are removed. The median nucleus area is defined for each sample image individually. Afterwards, the thyroid images and the thyroid segmentations are used to extract individual nucleus crops and nucleus masks.

When applying the feature-based classification to a new dataset, it has to be ensured that the image resolution of the new dataset is equal to the image resolution of the segmentation model training data. Otherwise the segmentation model has to be retrained on the desired image resolution or the image resolution of the new dataset has to be scaled.

#### Cell nucleus feature extraction and aggregation

For each segmented cell nucleus individual features are extracted. An abstraction of the process is depicted in the feature extraction part of Fig 2. The feature extraction for each nucleus is performed with the nucleus mask in combination with the corresponding nucleus in the microscopy color image and a grayscale version of the color image. For simplicity, this grayscale version of the microscopy images is not depicted in Fig 2.

Nuclear scoring by pathologists can be grouped into changes of size and shape, membrane irregularities and chromatin aspects. Our automated feature extraction selects other features, which do not necessarily belong to only one of the groups and can resemble many manual features at once. For example, texture features could implicitly assess many different membrane irregularity and chromatin distribution features. As such, for image processing, the features can be divided into the three category’s color, shape, and spatial features, respectively. All color features are listed in S1 Table in S1 Appendix, while all shape features are listed in S2 Table in S1 Appendix, and the spatial features are listed in S3 Table in S1 Appendix. For the color features, the RGB image and the grayscale image of each nucleus are evaluated. For the shape features, solely the nucleus segmentation is used (see Fig 2, nucleus masks). For the spatial features, solely the thyroid segmentation is used (see Fig 2, thyroid segmentations). The extracted features resemble the features used by pathologists for PTC-nuclear scoring [3].

*Color features*. The color features reassemble the membrane irregularities and chromatin characteristics addressed in [3]. Therefore, the mean and standard deviation of the nucleus color are calculated separately for each color channel (red r, green g, blue b) and the grayscale image of the nucleus are extracted. To focus on the features of intranuclear cytoplasmic inclusions, chromatin clearing, and margination to the nuclear membranes, the changes in color comparing the center, middle, and the border of the nucleus are calculated. This is achieved by creating three equally sized areas and calculating color ratios among them. Membrane irregularities and chromatin characteristics can also be represented by features distinguishing different texture patterns. Since gray level co-occurrence matrices (GLCMs) and their properties yielded good results in [6], we also use them together with the Shannon entropy in our study. In total, 23 color features are generated.

*Shape features*. The size and shape of nuclei are an important feature used by pathologists in thyroid PTC-nuclear scoring. The shape features, including area, perimeter, eccentricity, and solidity are extracted from the instance segmentation. The feature area refers to the number of pixels inside the instance segmentation for each nucleus. The eccentricity is measured by fitting an ellipse to each nucleus and calculating the eccentricity of the ellipse. The solidity is a measure for the distortion of the nucleus border and is calculated by the ratio of pixels inside the nucleus to the pixels of the convex hull. In total, four different shape features are calculated.

*Spatial features*. One of the features used by pathologists is nuclear crowding. This spatial feature refers to the number of neighborhood nuclei. We measure this by counting the number of nuclei in different radii around each nucleus. To define the radii, the mean cell radius *r*_*mean*_:

$$\begin{array}{c} r_{mean}=\frac{1}{n}\sum_{k=1}^{n}\sqrt{\frac{A_{k}}{\pi }}, \end{array}$$ (1)

where n is the total number of nuclei in the image and *A*_*k*_ is equal to the area of nucleus k, is calculated. The radii are afterwards calculated by *x* ⋅ *r*_*mean*_. Because we do not know which radii are most relevant for the classification into PTC-like and non-PTC-like, we use a large variety of different radii and therefore define *x* ∈ {3, 5, 7, 9, 15, 20, 25, 30}. A total of eight different distances are used to consider crowding in small and large areas. During the following feature selection step, the less relevant radii can be omitted. We do not perform any correction regarding nuclei at the borders of the images since our images are reasonably large. When applying the method to small images with a high border to center ratio, border corrections e.g. with reflection padding may be needed. Furthermore, the Euclidean distance to the nearest neighbor nucleus is calculated. In total, nine different spatial features are calculated.

*Sample aggregation*. Features are generated for each nucleus individually, for a total of 36 features. Each dataset in our experiments has more than 700,000 nuclei. While it is possible to classify each nucleus individually, this is computationally not feasible when combined with nested cross-validation. By sample aggregation, the number of samples in the dataset and therefore computational time is reduced. This is performed by representing the distribution of each feature for all nuclei of a patient by the mean and the standard deviation. However, this could possibly result in loss of useful information. Despite the mean and standard deviation, additional characteristics of the distribution could be used to describe it, but this increases the size of the final number of features and thus computational time. Using the mean and standard deviation results in 72 features for each patient, and the number of samples is reduced to the number of patients in the dataset. When multiple images are available for a patient, samples are merged across images and not individually for each image.

#### Preprocessing and classification

A large variety of different algorithms exist for data preprocessing and classification. To achieve the best results possible, the different algorithms have to be compared for each dataset since there is no way to know which combination will have the best results beforehand. For data preprocessing, we use quantile transformation and standard scaling. Each data preprocessing step can be applied or skipped. This results in different data preprocessing combinations, e.g., use of quantile transformation and no standard scaling. Furthermore, different feature selection methods are examined. As a filter based method, the univariate *χ*^2^ feature selection is used. Sequential forward selection from the MLxtend framework [17] is used as a wrapper-based method. Only one feature selection method is use at a time. Additionally, feature selection can be skipped. Most classifiers have hyperparameters which also have to be tuned for maximum performance.

This results in many hyperparameter combinations whereas thyroid datasets are often small. As a result, biased test performance can occur when cross-validation is used [18]. To reduce this bias, we use nested cross-validation in our experiments, where model fitting and hyperparameter selection is performed independently [18]. We split the data into five folds for the outer cross-validation and four folds are used for each inner cross-validation. Inside the inner cross-validation only the classifier hyperparameters are optimized, while a separate nested cross-validation is performed for each preprocessing, feature selection and classifier combination. It has to be noted, that not optimizing the data preprocessing hyperparameters, feature selection method and the chosen classifier inside the nested cross-validation could bias the result. We choose this trade-off to be able to discuss results like the used preprocessing method, the number of features selected and the used classifier in a much more intuitive manner. Furthermore, all splits are performed in a stratified manner regarding the distribution of the dataset into PTC-like and non-PTC-like.

### Deep learning-based classification

**Fig 4:**
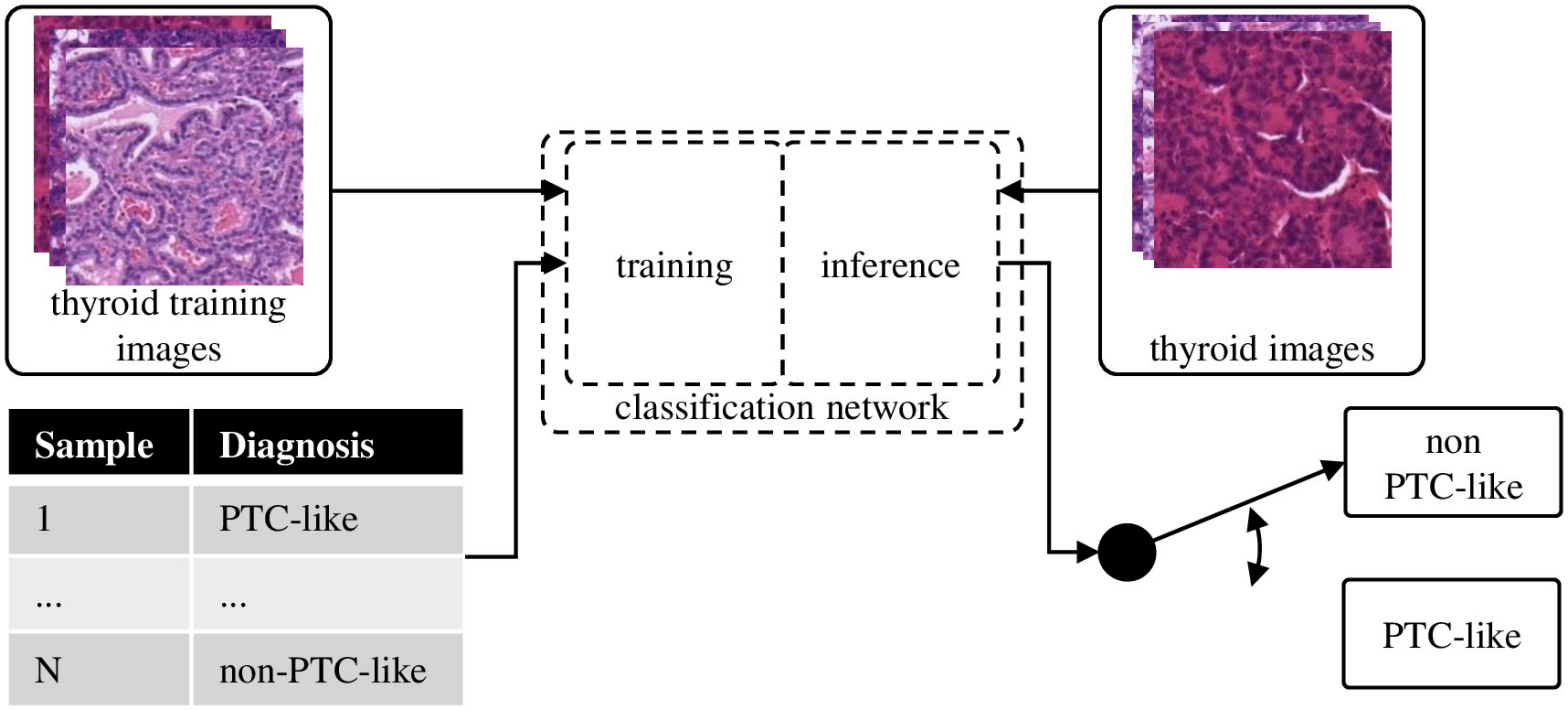
The deep learning-based classification performs the classification directly on the thyroid images.

The feature-based classification is compared to the direct classification with the deep learning-based classification. As shown in Fig 4, the different steps needed for the classification are significantly reduced. Therefore, the complexity of this approach is comparable to the segmentation step of the feature-based classification since we also utilize a convolutional neural network for this step. Time consuming feature engineering and setup of preprocessing pipelines is not needed. To improve performance and reduce training time on ImageNet [19], pretrained versions of the models are used. For all models investigated in the experiments, the last layer is replaced by a fully connected linear layer with two output neurons representing the PTC-like and non-PTC-like classes. The Adam optimizer [20] is used together with the cross-entropy loss. The training setup is further described in S1 Appendix.

We investigate different neural network and augmentation combinations specified in the Hyperparameter selection section. Due to the computationally intensive training of neural networks, cross-validation is used instead of nested cross-validation for the deep learning-based classification. To be able to compare the results to the feature-based classification, we perform a cross-validation, where the splits are the outer splits of the feature-based classification. The cross-validation is performed for each network and augmentation combination separately. Because we initialize the neural network weights randomly, we divide the training data for each split into 80% training and 20% validation data and train five models for each split. The best model for each split according to the validation data was applied to the corresponding test data afterwards. The final validation and test accuracy was the mean accuracy of the chosen models over the five splits.

When multiple images per sample are available in the test data, the final classification is determined by the majority. If the network’s prediction has a 50–50 ratio, the wrong class will be assumed for the sample.

### Hyperparameter selection

#### Feature-based classification

A large variety of algorithms have to be compared to identify the maximum quality achievable with feature-based classification. The algorithms itself can be represented by a hyperparameter. The data preprocessing hyperparameters are quantile transformation and standard scaling. Additionally, each feature selection method together with the number of selected features, ranging from 1 to 25 are defined as feature selection hyperparameters.

The six classifiers:

- Support Vector Classification (SVC),
- K-Nearest Neighbors (KNN),
- Gaussian Naive Bayes (GNB),
- Decision Tree (DT),
- Logistic Regression (LogReg), and
- Random Forest (RF)

are trained using Scikit-learn [21]. Each classifier has its individual set of classifier hyperparameters, which were optimized inside the nested cross-validation. The classifier hyperparameters used for each classifier are listed in S1 Appendix.

#### Deep learning-based classification

In the experiments, the residual neural networks ResNet18, ResNet50 and ResNet101 [22] are compared in combination with five different augmentation setups.

Augmentations were performed with the Albumentations framework [23]. Three handcrafted augmentations (*minimal*, *standard*, and *big*) were investigated. While *minimal* consists of a random cropping, flipping, and normalization, *standard* adds pixel level transformations like contrast limited adaptive histogram equalization, blur, or noise. The *big* augmentation setup adds Fourier Domain Adaptation to the standard augmentation [24]. This transfers the style of one image onto another image during training. Furthermore, two augmentations created with AutoAlbument [25] were investigated. This set of augmentations was picked to investigate whether complex augmentations help the networks to generalize better on the training data or prevent them from learning the relevant features. Furthermore, the AutoAlbument augmentations were picked to assess whether automated augmentation tools are superior to hand-crafted augmentation pipelines.

Additional training information and the augmentation setups can be seen in S1 Appendix.

## Results

### Overview

The results for the Tharun and Thompson dataset are shown first followed by the results for the Nikiforov dataset including the comparison to expert pathologists. Furthermore, we examine the domain gap between both datasets and the generalization ability of both methods in S1 Appendix.

#### Tharun and Thompson dataset

For the Tharun and Thompson dataset, the classification results of the feature-based classification are shown first. Afterwards, the influence of the hyperparameter optimization for the feature-based classification is investigated. The results for the deep learning-based classification are shown subsequently. At the end, the influence of the diagnostic groups present in the dataset is analyzed.

A full list of the trained feature-based classification models and the trained deep learning-based classification models together with the achieved accuracies on the Tharun and Thompson dataset is provided in S1 File.

#### Feature-based classification

For the feature-based classification, three hyperparameter combinations achieve the exact same accuracy on the validation data. SVC is used for all of them. Therefore we opted for the one with the least number of features used to achieve this result. The result is achieved by using quantile transformation and not applying a standard scaler for the data preprocessing. Additionally, sequential forward selection with a total of 22 features is used during training. The accuracy on the test data is 89.7% (Cohen’s Kappa 0.79). The classifier achieves an accuracy of 86.4% on the validation data and 97.4% on the training data.

**Fig 5:**
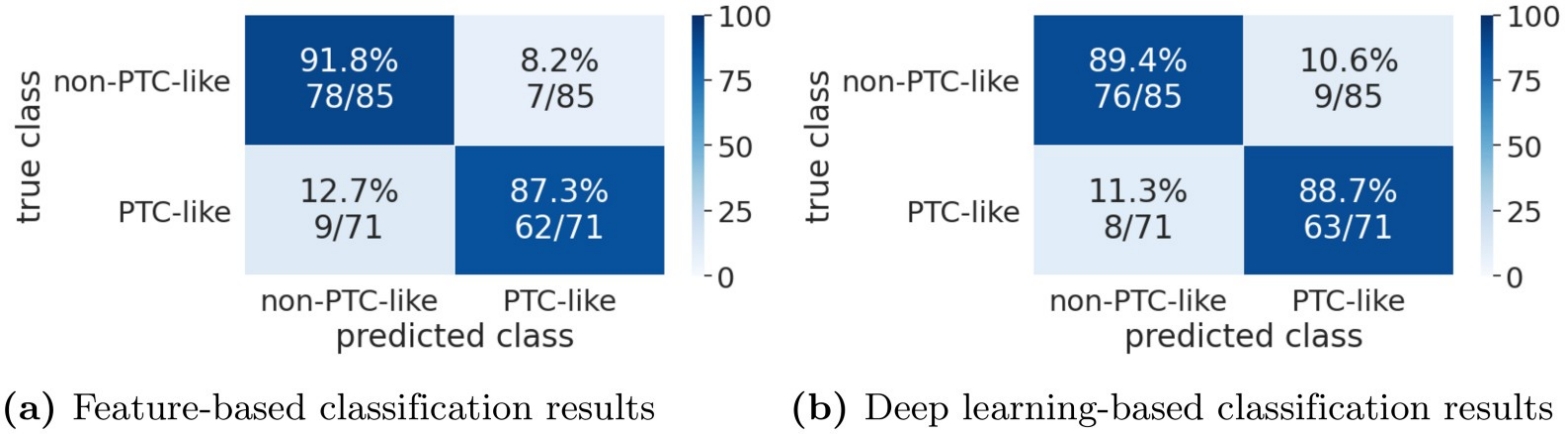
Confusion matrices for the Tharun and Thompson dataset.

A confusion matrix for the feature-based classification results is shown in Fig 5a.

**Fig 6:**
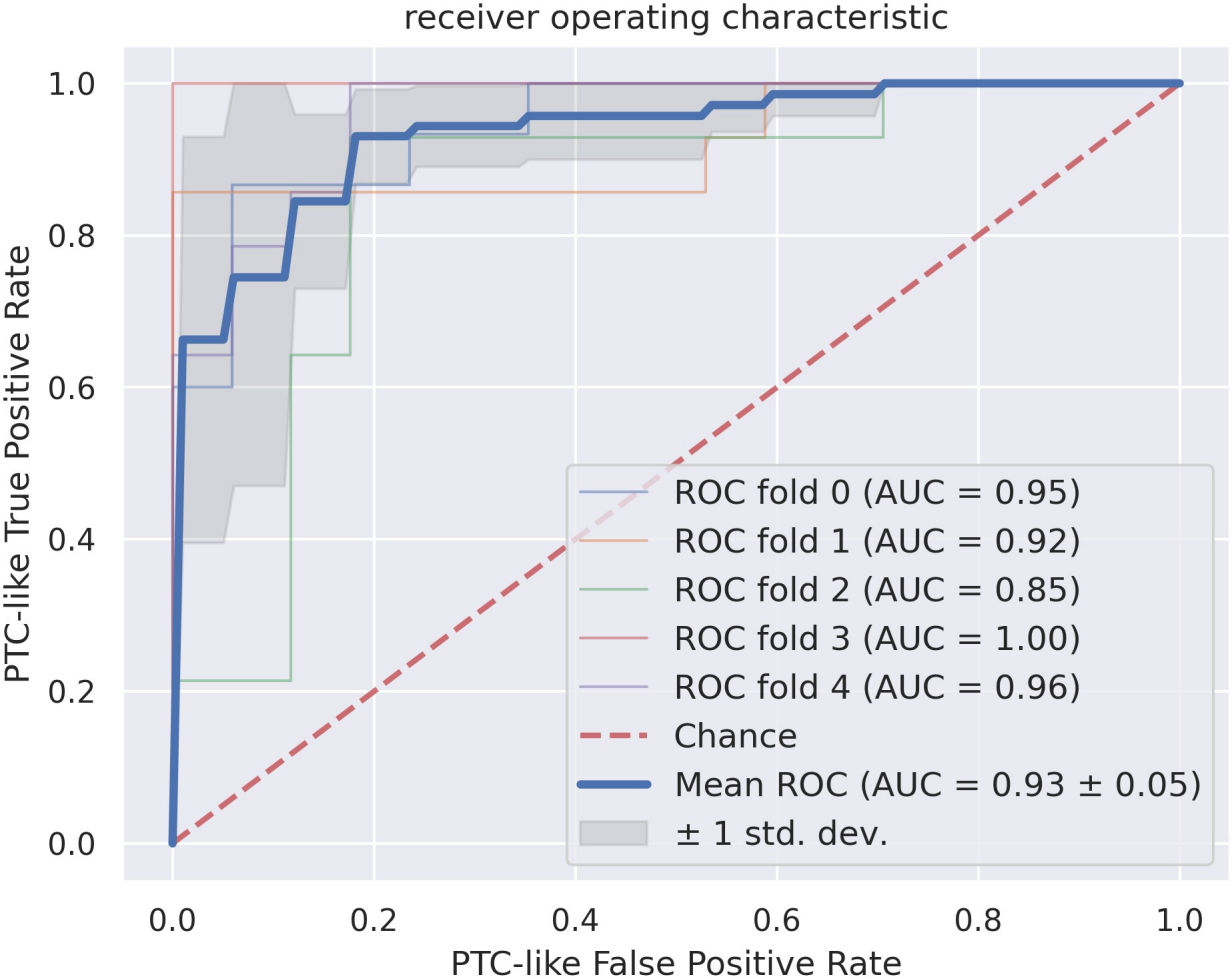
The receiver operating characteristic curves for the feature-based classification, where a true positive sample is a PTC-like sample classified as PTC-like. All cross-validation splits and the resulting area under curve (AUC) together with the resulting mean for the dataset are shown. The standard deviation around the mean is annotated in dark gray. The red dotted line corresponds to the classification by chance.

The receiver operating characteristic (ROC) curves for the feature-based classification are shown in Fig 6. The mean ROC curve of the cross-validation splits has an area under curve (AUC) of 0.93 ± 0.05. Thus, the standard deviation is low. The ROC curve for the ROC fold 2 split is notably worse then the ROC curves of the other splits.

To analyze what features are the most important, we trained the final classifier on the whole dataset. Since sequential forward selection was used for feature selection, we used it to extract the features selected first. The five most import features are the mean of the nuclei solidity, standard deviation of change of color comparing the nucleus border to the middle, mean of the GLCM correlation feature, the mean of the nucleus area, and the mean of the mean nucleus color in the green color channel. Therefore, color features as well as shape features are in the five most important features, while no spatial features are present.

#### Feature-based hyperparameter optimization

To assess the effect of hyperparameter tuning, we trained a baseline model and compared the performance to the tuned model. For the baseline model, we used SVC with standard scikit-learn hyperparameters. Therefore, hyperparameter optimization inside the nested cross-validation has been disabled, while the outer splits of the nested cross-validation stayed the same, resulting in common cross-validation. Standard scaling is applied and no feature selection is used. This baseline model achieved an accuracy of 85.9% and therefore hyperparameter optimization results in a classification improvement of 3.8%.

Additionally, we examined whether the same preprocessing, feature selection method and classifier work on different datasets. We extracted the best hyperparameter setting on the Nikiforov dataset (quantile transformation, standard scaling and *χ*^2^ feature selection with a total of 22 selected features) and used it for the nested cross-validation on the Tharun and Thompson dataset. This results in an accuracy of 84.0% on the test data. Using the Nikiforov dataset hyperparameters on the Tharun and Thompson dataset thus results in an accuracy decrease of 1.9% compared to the baseline model and 5.7% compared to the model with hyperparameters optimized on the Tharun and Thompson dataset.

#### Deep learning-based classification

For the deep learning-based classification, the best model achieves an accuracy of 89.1% on the test data (Cohen’s Kappa 0.78), right at the transition from a moderate to a strong level of agreement [26]. It achieves an accuracy of 94.5% on the validation data. A ResNet101 with the *big* augmentation is used. A confusion matrix for the deep learning-based classification results is shown in Fig 5b.

#### Diagnostic group evaluation

To evaluate the influence of the diagnostic groups present in the dataset, the results were split into their initial diagnostic groups in Table 1. The classifiers performs worst for the NIFTP (PTC-like) group. The NIFTP samples have the smallest nuclear alterations compared to FVPTC and CPTC, with only nine NIFTP samples in the dataset. CPTC has the strongest nuclear alterations and is classified with 91% and 94% accuracy. FTC and FA both have no PTC-like nuclear alterations, with the sample size and accuracy for FA higher than for FTC for both classifiers.

**Table 1:** Number of correct predictions relative to the amount of samples split into the diagnostic groups of the initial dataset. Results are shown for the feature-based classification (FBC) and deep learning-based classification (DLC).

diagnosis	FBC	DLC
FTC	26/32 (81%)	27/32 (84%)
FA	52/53 (98%)	49/53 (92%)
NIFTP	6/9 (67%)	5/9 (56%)
FVPTC	8/9 (89%)	8/9 (89%)
CPTC	48/53 (91%)	50/53 (94%)

### Nikiforov dataset

Also for the Nikiforov dataset, the classification results of the feature-based classification are shown first, followed by the influence of the hyperparameter optimization. After that, the deep learning-based classification results are shown. Because the classification by 24 expert pathologists was present for the Nikiforov dataset, we were able to conduct an in depth comparison of the methods to expert pathologists. A full list of the trained feature-based classification models and the trained deep learning-based classification models together with the achieved accuracies on the Nikiforov dataset is provided in S2 File.

#### Feature-based classification

The accuracy for the feature-based classification on the test data is 83.5% (Cohen’s Kappa 0.46). The classifier achieves 85.9% on the validation data and 90.4% on the training data. This is achieved by using quantile transformation and standard scaling. For feature selection, *χ*^2^ feature selection with a total of 22 selected features is used. The classifier utilized is SVC.

**Fig 7:**
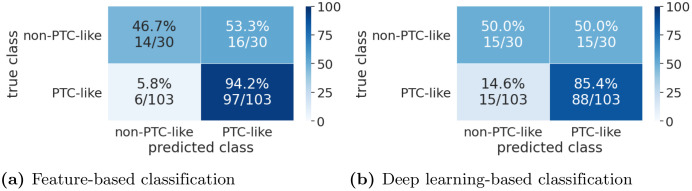
Confusion matrices for the Nikiforov dataset.

Confusion matrices for the results are shown in Fig 7a. The PTC-like samples are classified with a much higher accuracy than the non-PTC-like samples. This could be partially due to the dataset distribution, since 77% of the samples have PTC-like features and stratified cross-validation is used. Therefore, it is beneficial for the classifiers to classify uncertain samples as PTC-like.

**Fig 8:**
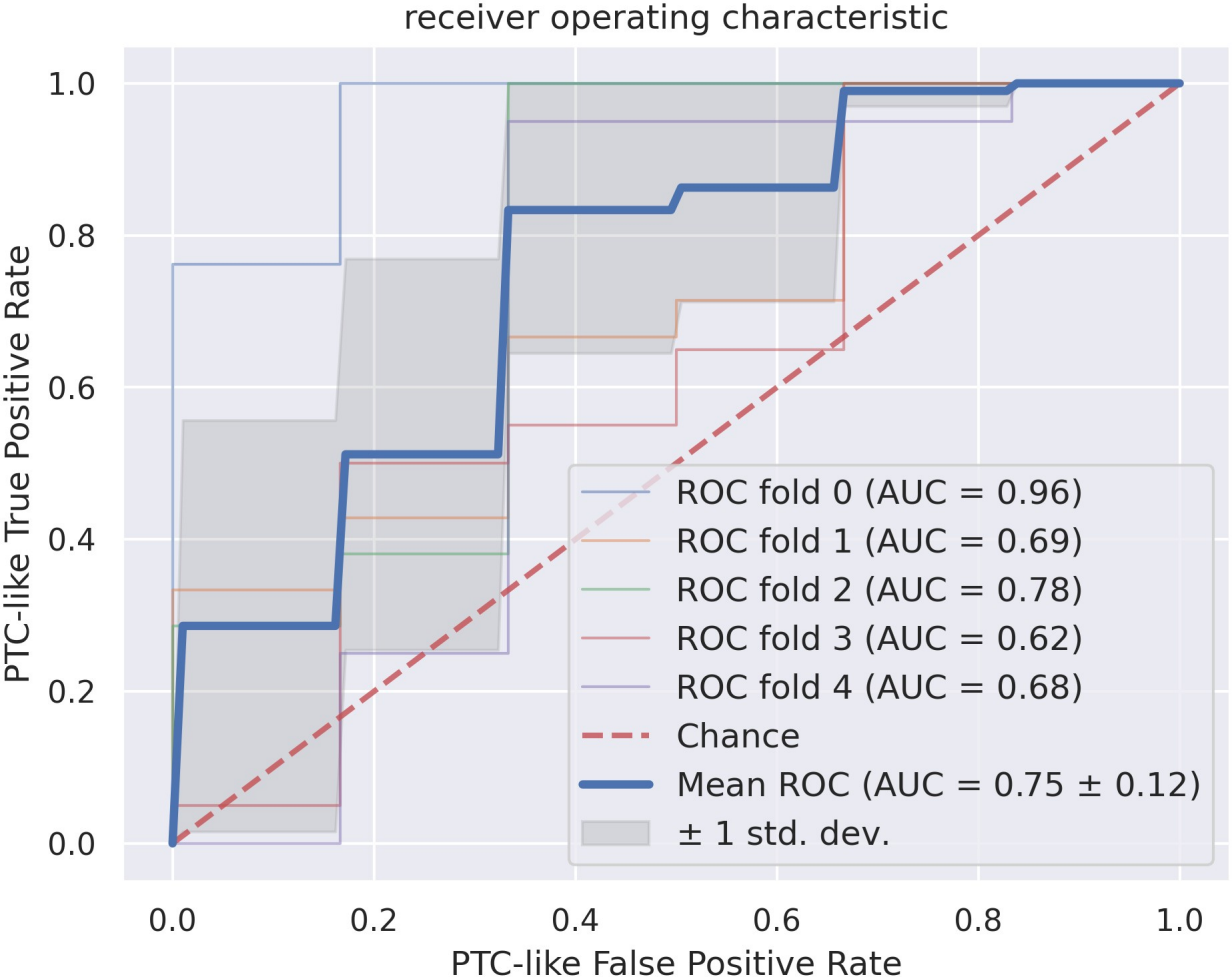
The receiver operating characteristic curves for the feature-based classification, where a true positive sample is a PTC-like sample classified as PTC-like. All cross-validation splits and the resulting area under curve (AUC) together with the resulting mean for the dataset are shown. The standard deviation around the mean is annotated in dark gray. The red dotted line corresponds to the classification by chance.

The ROC curves are shown in Fig 8. The mean ROC curve of the cross-validation splits has an AUC of 0.75 ± 0.12. Thus, the standard deviation is high and the results for the splits differ considerably.

Finally, we analyzed which features are the most important for the feature-based classification on the Nikiforov dataset. Since the features are selected by *χ*^2^ feature selection, no classifier had to be trained. The five most important features regarding *χ*^2^ feature selection on the whole dataset are: the standard deviation of the perimeter, the standard deviation of the area, mean of change of color comparing the nucleus border to the middle, mean change of color comparing the nucleus border to the center, and the standard deviation of the standard deviation of the mean nucleus color in the green color channel. Therefore, similarly for the Nikiforov dataset, color and shape features are in the five most important features, while no spatial features are present.

#### Feature-based hyperparameter optimization

We also assessed the effect of hyperparameter optimization on the Nikiforov dataset. The baseline model is the same as for the Tharun and Thompson dataset. The baseline model trained on the Nikiforov dataset achieves an accuracy of 79.6%, decreasing the accuracy by 3.9% compared to the model with optimized hyperparameters.

Additionally, we investigated, whether the preprocessing, feature selection method and classifier performing best on the Tharun and Thompson dataset (quantile transformation, no scaling, sequential forward selection with 22 selected features and SVC) also yield good performance on the Nikiforov dataset. Therefore, we used this setup for nested cross-validation on the Nikiforov dataset. The resulting accuracy is 80.5%, decreasing the accuracy by 3.0% compared to the model with optimized hyperparameters.

#### Deep learning-based classification

For the deep learning-based classification, the best performing model is a ResNet101 with the *standard* augmentation. The model achieves an accuracy of 77.4% on the test data (Cohen’s Kappa 0.35) and 88.9% on the training data. It performs 6.1% worse than the feature-based classification.

#### Expert pathologist comparison

In addition to the ground truth classification, the Nikiforov dataset was classified by an international panel of 24 thyroid gland expert pathologists. For each sample, the number of pathologists classifying the sample as PTC-like and non-PTC-like was used. With this data, we were able to conduct an in depth comparison of both methods to the expert pathologists. To do so, the mean expert pathologist rating *c*_*i*_ for each sample *i* can be calculated by:

$$\begin{array}{c} c_{i}=\frac{\text{max}(c_{i}^{n},c_{i}^{p})}{c_{i}^{n}+c_{i}^{p}}, \end{array}$$ (2)

where $c_{i}^{n}$ is the number of pathologists grading the sample *i* as non-PTC-like and $c_{i}^{p}$ is the number of pathologists grading the sample *i* as PTC-like. The mean expert pathologist rating for the whole dataset was obtained by calculating the mean of *c*_*i*_ which is equal to 71.2% and therefore lower than the accuracy of both classifiers.

Furthermore, it can be calculated how the classifiers perform on samples where the pathologists dissent in grading and therefore *c*_*i*_ is small and how they perform on samples where the pathologists agree upon and therefore *c*_*i*_ is close to 100%. This is undertaken by evaluating not the whole dataset, but only samples where *c*_*i*_ is above specific thresholds (the minimal pathologist agreement level). As an example, all results could be calculated only for samples where *c*_*i*_ is ≥ 80%. A total of 27 samples remain for evaluation, where 25 samples are PTC-like and 2 samples are non-PTC-like resulting in 92.6% of the samples being PTC-like. The mean expert pathologist rating for this samples rises to 87.6%. The feature-based classification achieves 100% on the remaining samples, while the deep learning-based classification achieves 96.3%.

**Fig 9:**
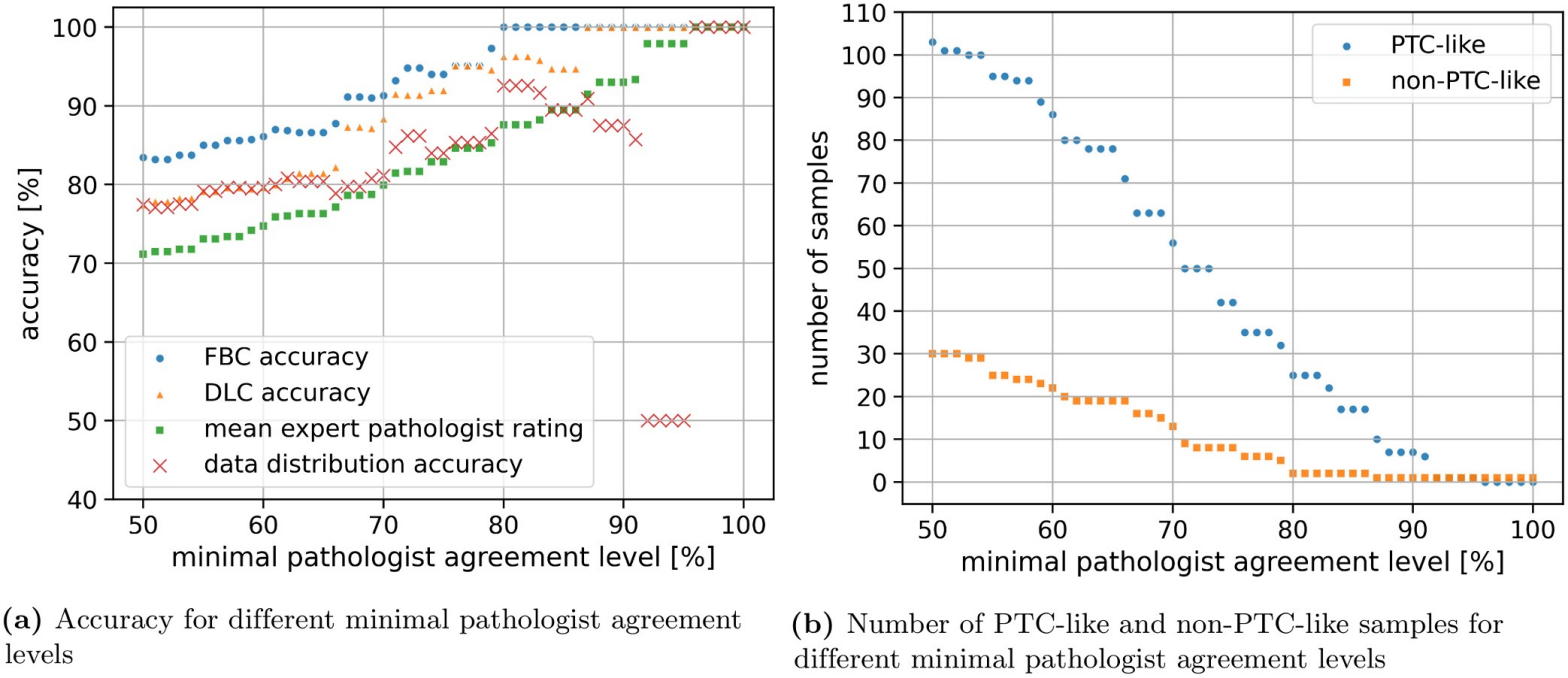
The evaluation on samples with a pathologist agreement level greater than a threshold is shown in (a). The feature-based classification (FBC accuracy, blue dots) outperforms the mean expert pathologist rating (green squares) and the accuracy achievable by knowing the data split (red crosses). The performance of the deep learning-based classification (DLC accuracy, orange triangles) varies between the FBC accuracy and the mean expert pathologist rating. The number of samples evaluated for different minimal pathologist agreement levels is shown in (b). The PTC-like samples are shown in blue dots and the non-PTC-like samples are shown in orange squares.

An evaluation on samples where *c*_*i*_ is equal or greater than different thresholds is shown in Fig 9. The accuracy of the feature-based classifier surpasses 90% (Cohen’s Kappa 0.67) when being evaluated on samples with *c*_*i*_ ≥ 67%. The classifier already reached 100% when being evaluated on samples with *c*_*i*_ ≥ 80%.

Additionally, the data distribution accuracy is shown in Fig 9, which refers to the accuracy achievable by only knowing the class distribution of the data. For *c*_*i*_ ≥ 80% there are 63 PTC-like samples and 16 non-PTC-like samples. Therefore, 79.7% accuracy is achievable by classifying all samples as PTC-like. The feature-based classifier always outperforms the accuracy achievable from just knowing the data distribution.

## Discussion

The experimental results show that feature-based classification is still able to outperform a deep learning-based classification. However, the creation of the feature-based classification is more time consuming. Although the problem setting is the same, the best data preprocessing and feature selection methods differ for both datasets. To achieve the best performance on new datasets, it is necessary to conduct a computational intensive hyperparameter search, which is not feasible for many clinics.

As the Nikiforov dataset contains mostly borderline tumors, many of which were reclassified as NIFTP, known to show less distinct PTC-like nuclear features, both methods performed worse on this dataset. Thus, performance of both methods on new data has to be verified. If the performance significantly differs from the expected performance, the algorithms may have to be individually trained on the new data before being used in clinical practice.

Furthermore, it must be assumed that on the available datasets both methods are not able to reliably detect borderline samples, i.e., NIFTP samples. Nevertheless, both methods are able to classify FA and CPTC samples with a very high accuracy. During the creation phase of new datasets, challenging borderline samples must be included, otherwise the classifications will result in unrealistically high accuracies that would not transfer to clinical practice. Comparing the results of both methods on the Nikiforov dataset to the classifications of the expert pathologists supports this theory, since samples that are difficult to classify by humans are also the most challenging for automated methods.

Both compared methods can be used to assist pathologists in their daily work, while the feature-based classification has the advantage of providing the features as additional data for the pathologists. This could lead to greater acceptance by pathologists and thereby lead to improved diagnostic accuracy while reducing their workload. Ways to incorporate these methods into their daily working routines requires further investigation.

Notably, the newly created features representing color changes inside the nucleus were present in the five most important features for both datasets. This suggests that features like chromatin clearing, glassy nuclei, and chromatin margination to the nuclear membrane used by pathologists are represented by these chromatin features. It is of additional interest that these features are the ones used by expert pathologists to the highest kappa reproducibility of 0.68 (mean; median 0.67; SD 0.14), than what was achieved based on size and shape (0.54; median 0.60; SD 0.19) and membrane irregularities (0.61; median 0.67 SD 0.26) [3]. Thus, the spatial features able to detect crowding of nuclei had no additional information important enough to be in the top five features.

The deep learning-based classification performs not as well as the feature-based classification on the Nikiforov dataset, while it yields a nearly identical performance on the Tharun and Thompson dataset. One explanation could be the properties of the Nikiforov dataset. The Nikiforov dataset only contains cases initially classified as FVPTC-like. Reclassification by an expert panel removed 30 cases into a non-PTC-like category. Therefore, one can conclude that the non-PTC-like cases are all difficult, borderline cases at the transition between non-PTC-like to PTC-like tumors. It seems the deep learning-based method is not able to draw a boundary between these cases correctly. For the feature-based classification on the Nikiforov dataset, the shape features perimeter and nucleus area are the most important features. Therefore, especially for borderline cases, shape features are the most relevant features and the feature-based classification is able to explicitly use this information for decision making. Moreover, the Nikiforov dataset could be too small for the use of the deep learning-based method with only 79 tumors in the training data.

## Conclusion

Automated thyroid tumor classification using nuclear alterations is a challenging task. Incorporation of machine learning methods into the clinical routine could possibly reduce the workload of pathologists and decrease inter-observer variability. To evaluate the applicability of machine learning methods, we compared feature-based classification and deep learning-based classification with respect to their ability to classify thyroid gland tumors into PTC-like and non-PTC-like on two datasets.

In our experiments on the Nikiforov dataset, the feature-based classification achieves an accuracy of 83.5% (Cohen’s Kappa 0.46). It performs 6.1% better than the deep learning-based classification and exceeds the mean expert pathologist rating. For the Tharun and Thompson dataset, the feature-based classification achieves an accuracy of 89.7% (Cohen’s Kappa 0.79) exceeding the deep learning-based classification by 0.6%.

High accuracies are achieved by both methods while no method was clearly superior in all aspects. We advise to use the feature-based method on small datasets with many borderline cases. When applying the methods to new unseen datasets, it should be assessed whether they are able to generalize on this data. The accuracies achievable are comparable to expert pathologists and can provide decision support through a second opinion.

To further increase the classification performance we see a need for bigger datasets especially incorporating borderline cases, since neural networks often benefit a lot from large datasets.

In order to further improve usability of machine learning-based methods for pathologists, we want to incorporate frameworks explaining why predictions are made in the future [27, 28]. Additionally we would like to extend the feature-based classification to incorporate other nuclear features, such as the rate of nuclei containing perinucleolar halos. Preliminary data of ours and a study by Suzuki, et al. [29], shows that this might be a feature more frequent in PTC-like nuclei, but automated detection of perinucleolar halos still poses a technical challenge.

## Supporting information

S1 Appendix Further methodical information. Further information regarding the feature-based classification and deep learning-based classification methods.
(PDF)

S1 Fig Example images. Example images from the Nikiforov dataset and the Tharun and Thompson dataset from both classes PTC-like and non-PTC-like.
(PNG)

S1 File Tharun and Thompson results. Full list of classification results on the Tharun and Thompson dataset for all trained feature-based classification and deep learning-based classification variants.
(XLSX)

S2 File Nikiforov results. Full list of classification results on the Nikiforov dataset for all trained feature-based classification and deep learning-based classification variants.
(XLSX)
