# Supplementary material for: Machine learning methods for automated classification of tumors with papillary thyroid carcinoma-like nuclei: A quantitative analysis
Source: PLoS One. 2021 Sep 22;16(9):e0257635. doi: 10.1371/journal.pone.0257635 (PMC8457451; doi:10.1371/journal.pone.0257635)
Supplement: S1 Appendix — Further information regarding the feature-based classification and deep learning-based classification methods. (PDF) [file pone.0257635.s001.pdf]

Machine learning methods for automated classification of tumors with papillary thyroid carcinoma-like nuclei: a quantitative analysis

S1 Appendix: Additional information regarding cell nuclei feature extraction, the deep learning-based classification, hyperparameter selection, the domain gap and generalization.

Moritz Böhland, Lars Tharun, Tim Scherr, Ralf Mikut, Veit Hagenmeyer, Lester Thompson, Sven Perner, Markus Reischl

Cell nuclei feature extraction

Tables S1, S2 and S3 shows the feature categories color, shape and spatial and the features extracted for each category in combination with the corresponding medical features and a short feature description.

Table S1. Medical features and the corresponding color features created together with a short feature description. The *ratio*, $GLCM$, and *shannon* features are calculated solely on the grayscale version of the image.

medical features	color features	feature description
irregular contours, grooves, folds, intranuclear cytoplasmic inclusions, chromatin clearing, margination to the nuclear membranes, glassy nuclei	r_{mean}<br>g_{mean}<br>b_{mean}<br>$gray_{mean}$	mean of the pixel value inside the nucleus for the red (r), green (g), blue (b) color channel, and the grayscale image (gray)
	r_{std}<br>g_{std}<br>b_{std}<br>$gray_{std}$	standard deviation of the pixel value inside the nucleus for the red (r), green (g), blue (b) color channel, and the grayscale image (gray)
intranuclear cytoplasmic inclusions, chromatin clearing, margination to the nuclear membranes	$ratio^{mean}_{BM}$<br>$ratio^{mean}_{BC}$<br>$ratio^{mean}_{MC}$	separate mean intensity for pixels in the nucleus middle (M), center (C) and border (B) and calculation of ratios afterwards
	$ratio^{std}_{BM}$<br>$ratio^{std}_{BC}$<br>$ratio^{std}_{MC}$	separate standard deviation of intensity for pixels in the nucleus middle (M), center (C) and border (B) and calculation of ratios afterwards
membrane irregularities and chromatin characteristics, grooves, Chromatin clearing, margination to the nuclear membranes, glassy nuclei	$GLCM^{1}_{energy}$<br>$GLCM^{2}_{energy}$<br>$GLCM^{1}_{homogeneity}$<br>$GLCM^{2}_{homogeneity}$<br>$GLCM^{1}_{dissimilarity}$<br>$GLCM^{2}_{dissimilarity}$<br>$GLCM^{1}_{correlation}$<br>$GLCM^{2}_{correlation}$	texture pattern features by calculation of gray level co-occurrence matrices for the pixel distances 1 and 2
	shannon	texture pattern feature by calculation of Shannon entropy

Table S2. Medical features and the corresponding shape features created together with a short feature description.

medical features	shape features	feature description
Enlargement, elongation, irregular contours	*area*<br>*eccentricity*<br>*perimeter*<br>*solidity*	features in respect to binary pixel level segmentation of the nucleus

Table S3. Medical features and the corresponding spatial features created together with a short feature description.

medical features	spatial features	feature description
crowding	$neighbor_{dist}$	distance to the center of the nearest nucleus
	$neighbors_3$<br>$neighbors_5$<br>$neighbors_7$<br>$neighbors_9$<br>$neighbors_{15}$<br>$neighbors_{20}$<br>$neighbors_{25}$<br>$neighbors_{30}$	Number of nuclei in a circle around a nucleus

Color features For each color channel (r,g,b) the mean and standard deviation (std) for all pixels inside each individual segmented cell nucleus are calculated (r_{mean}, r_{std}, g_{mean}, g_{std}, b_{mean}, b_{std}). In addition, the mean and standard deviation of each grayscale cell nucleus are calculated ($gray_{mean}$, $gray_{std}$).

The intranuclear cytoplasmic inclusion, chromatin clearing and margination to the nuclear membranes are detected by calculating relations between the local coloring of the nucleus. To do so, the RGB image of the nucleus is converted to a grayscale image. As seen in Figure S1 the nucleus shown in (a) has a darker border compared to the inside of the nucleus. To quantify this feature, the ratio between the border and the center or middle areas of the nucleus is calculated. The areas shown in (c) are calculated from the binary segmentation (b). This is done by calculating the Euclidean distance map of the segmented nucleus. Afterwards the pixel values of the distance map are grouped into three zones (border (b), center (c) and middle (m)) by using the 33% and 66% percentiles (excluding the value 0 outside the cell). Each area, depicted in (c) as white, light gray and dark gray has the same amount of pixels inside. The pixels of the grayscale nucleus image (a) inside each area (c) are reduced by calculating the mean and standard deviation. This results in the auxiliary features $z_b^{mean}, z_b^{std}, ..., z_m^{std}$ not used directly as classification features. The auxiliary features are used to calculate the relations between the local coloring separately for the mean and standard deviation auxiliary features with: $ratio_{BM} = z_b/(z_b + z_m)$, $ratio_{BC} = z_b/(z_b + z_c)$ and $ratio_{MC} = z_m/(z_m + z_c)$. The combinations $ratio_{CB}$, $ratio_{MB}$ and $ratio_{MC}$ are redundant to the existing features (e.g. $ratio_{BC} = 1 - ratio_{CB}$) and therefore not included in the feature set. Using mean and std for each region and the introduced ratios results in six local coloring relation features.

The features incorporating texture patterns are the gray level co-occurrence matrix features ($GLCM$). The GLCMs are calculated from pixels inside a rectangular bounding box containing the grayscale image of a nucleus. The GLCMs are calculated for the angles 0°, 45°, 90°, 135°. Since cell nuclei appear in all orientations and the cell orientation is no meaningful feature, the GLCMs for all angles are added together for each cell before calculating the texture properties. This can be interpreted as increasing cell sample size by rotating the cell nucleus. The pixel distances 1 and 2 are used. From the GLCMs the properties dissimilarity, correlation, energy and homogeneity are calculated [1]. By combining the texture

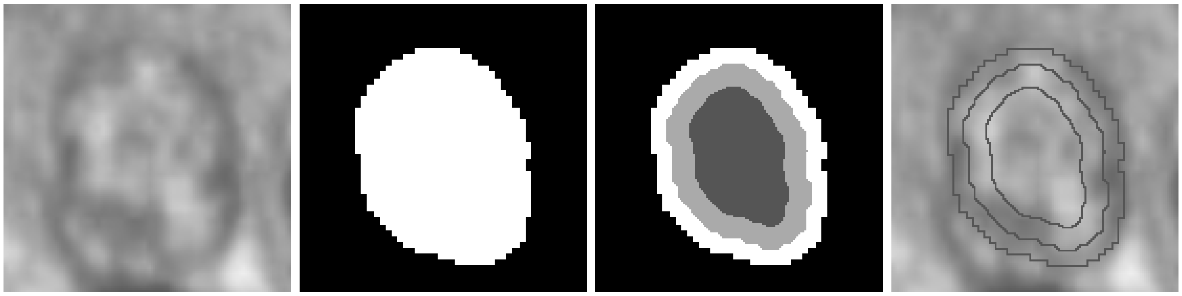

(a) nucleus **(b)** binary **(c)** areas **(d)** overlay

Fig S1. A magnification of a nucleus is shown in (a). The according binary segmentation is shown in (b). The segmentation is split into three areas with equal pixel size (c). An overlay of the areas is shown in (d). Color ratios of the different areas can afterwards be calculated.

properties with the pixel distances, eight GLCM features are created. Furthermore, the Shannon entropy for the grayscale image of the pixels inside a rectangular bounding box containing the cell nucleus is calculated [2]. In total, 23 color features are generated.

Shape features The shape features are extracted solely from the instance segmentation. The *area* feature refers to the amount of pixels inside the instance segmentation for each nucleus. Additionally, the *perimeter* can also be calculated directly from the instance segmentation. For the *eccentricity*, the ratio of focal distance over the major axis length of an ellipse with the same second-moments as the nucleus is calculated. Therefore, eccentricity is in the interval $[0, 1)$ where 0 represents a circle. The last shape feature is the *solidity*. Solidity is calculated by the ratio of pixels inside the nucleus to the pixels inside the convex hull of the nucleus. In total, four shape features are calculated.

Spatial features For the spatial features, an example image is shown in Figure S2, where the centroid for each nucleus is marked by a red dot. Exemplary, the three radii $x * r_{mean}$ for $x \in \{3, 5, 7\}$ are shown by red circles. Crowding of nuclei will result in a larger number of nuclei in the inner circles.

Deep learning-based classification

The deep learning-based classification is implemented in PyTorch Lightning utilizing the classification models provided with torchvision. To improve performance and reduce training time, the on ImageNet pretrained versions of the models are used. The last layer is replaced by a fully connected linear layer with two output features corresponding to the two classes PTC-like and non-PTC-like. The Adam optimizer is used in combination with cross-entropy loss. The initial learning rate is set by the PyTorch Lightning learning rate finder using the findings of. The learning rate is halved, if the loss on the validation data did not improved over the last 25 epochs. If the loss on the validation data did not improve for 50 epochs, training is stopped. The weights of the epoch with the best performance regarding the loss on validation data is chosen as the final model.

To compare the results to the feature-based classification, the outer splits of the nested cross-validation from the feature-based classification are used as cross-validation splits for the deep learning-based classification. The first inner split of the nested cross-validation is used for the training and validation split of the deep learning-based classification. Therefore, the test data is exactly the same for each split in both methods. Inside each split, five models are trained and the best performing one on the validation data is chosen as a final model for the split. The final validation and test accuracy is the mean accuracy of the chosen models.

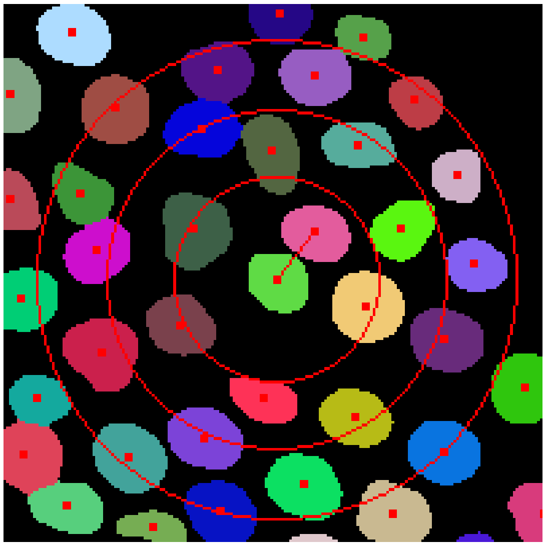

Fig S2. Crop sized 200x200 px of instance segmentation for patient 30 from the Tharun and Thompson dataset. The centroid for each nucleus is indicated by a red dot. The spatial features are shown for the green nucleus in the middle. The distance to the nearest neighbor nucleus is indicated by a red line. The radii $x * r_{mean}$ for $x \in \{3, 5, 7\}$ are shown. This results in 4, 10 and 23 neighbor nuclei.

The models are trained on random crops 512x512 px and the batch-size for ResNet18 and ResNet50 is set to 32. The batch-size for ResNet101 is set to 20. This enables training on 16GB VRAM.

Hyperparameter selection

Feature-based classification

Each classifier used in the nested cross-validation has it's own set of classifier hyperparameters. For SVC, the kernel (linear, polynomial of degree three) and the regularization parameter C (0.5, 1.0, 2.0) are optimized. Because the SVC did not converge for data preprocessing hyperparameter combinations with no quantile transformation and no standard scaling (102 data preprocessing hyperparameter combinations), this combinations are excluded for the optimization of the SVC. For KNN, the number of neighbors (2, 5, 10), the algorithm (BallTree, KDTree) and the leaf size (15, 30, 60) are optimized. For GNB and DT, no hyperparameters are optimized. The regularization hyperparameters C for LogReg is set to 0.5, 1.0, 2.0 and the maximum number of iterations is fixed to 1000. The number of estimators for RF is optimized for 2, 5, 10, 50, 100, 200.

A grid search for all possible combinations of data preprocessing hyperparameters, used feature selection method together with the number of features to select and the used classifier results in 2346 different training setups. The best performing one on the validation data is the final model and the accuracy on the test data is regarded as the final accuracy.

Deep learning-based classification

For the deep learning-based classification, different neural networks and different augmentation setups are defined as hyperparameters. For all augmentation setups, the images are random cropped and normalized. In addition, the minimal augmentation consists of a horizontal flip and 90° rotation. Both are applied with 50% likelihood. The standard augmentation adds one of the augmentations Contrast Limited Adaptive Histogram Equalization (CLAHE), change in contrast and change of brightness to the

minimal augmentations and combines it with blur and Gaussian noise. The big augmentations adds Fourier Domain Adaptation to the standard augmentation.

The code for the minimal augmentation is:

```
import albumentations as A
from albumentations.pytorch import ToTensorV2
t = A.Compose([
        A.RandomCrop(512, 512),
        A.Flip(),
        A.Normalize(mean=(0.485,0.456,0.406), std=(0.229,0.224,0.225)),
        ToTensorV2()])
```

and the code for the standard augmentation is:

```
import albumentations as A
from albumentations.pytorch import ToTensorV2
t = A.Compose([
        A.Flip(p=0.5),
        A.Rotate(p=0.5),
        A.RandomCrop(512, 512, always_apply=True),
        A.OneOf([A.CLAHE(p=0.33),
                 A.RandomContrast(p=0.33),
                 A.RandomBrightnessContrast(p=0.33)
                ], p=0.5),
        A.Blur(p=0.25),
        A.GaussNoise(p=0.25),
        A.Normalize(mean=(0.485,0.456,0.406), std=(0.229, 0.224, 0.225)),
        ToTensorV2())])
```

The code for the big augmentation is:

```
import albumentations as A
from albumentations.pytorch import ToTensorV2
t = A.Compose([
        A.Flip(p=0.5),
        A.Rotate(p=0.5),
        A.RandomCrop(512, 512, always_apply=True),
        A.domain_adaptation.FDA(train_images, beta_limit=0.05, p=0.5),
        A.OneOf([A.CLAHE(p=0.33),
                 A.RandomContrast(p=0.33),
                 A.RandomBrightnessContrast(p=0.33)
                ], p=0.5),
        A.Blur(p=0.25),
        A.GaussNoise(p=0.25),
        A.Normalize(mean=(0.485,0.456,0.406),
                    std=(0.229, 0.224, 0.225)),
        ToTensorV2())])
```

while all images in the training set are present in train_images.

Furthermore, two augmentation settings using AutoAlbument from the Albumentations framework are compared. The settings for the search.yaml file for the AutoAlbument setting one are:

```
task: classification
policy_model:
  num_sub_policies: 20
  num_chunks: 8
```

```
  operation_count: 4
classification_model:
  num_classes: 2
  architecture: resnet18
  pretrained: False
data:
  dataset:
    _target_: dataset.SearchDataset
  preprocessing:
   - RandomCrop:
        height: 512
        width: 512

  normalization:
    mean: [0.485, 0.456, 0.406]
    std: [0.229, 0.224, 0.225]
  dataloader:
    _target_: torch.utils.data.DataLoader
    batch_size: 32
    num_workers: 4
    shuffle: True
optim:
  epochs:  60
  main:
    _target_: torch.optim.Adam
    lr: 1e-3
    betas: [0, 0.999]
  policy:
    _target_: torch.optim.Adam
    lr: 1e-3
    betas: [0, 0.999]
hydra:
  run:
    dir: ${config_dir:}/outputs/${now:%Y-%m-%d}/${now:%H-%M-%S}
```

and the AutoAlbument settings two are:

```
task: classification
policy_model:
  num_sub_policies: 4
  num_chunks: 4
  operation_count: 4
classification_model:
  num_classes: 2
  architecture: resnet18
  pretrained: False
data:
  dataset:
    _target_: dataset.SearchDataset
  preprocessing:
   - RandomCrop:
        height: 512
        width: 512
```

```
  normalization:
    mean: [0.485, 0.456, 0.406]
    std: [0.229, 0.224, 0.225]
  dataloader:
    _target_: torch.utils.data.DataLoader
    batch_size: 32
    num_workers: 4
    shuffle: True
optim:
  epochs: 60
  main:
    _target_: torch.optim.Adam
    lr: 1e-3
    betas: [0, 0.999]
  policy:
    _target_: torch.optim.Adam
    lr: 1e-3
    betas: [0, 0.999]
hydra:
  run:
    dir: ${config_dir:}/outputs/${now:%Y-%m-%d}/${now:%H-%M-%S}
```

Both AutoAlbument setups are trained on the whole dataset. This could possibly leak test data into the training process. Since none of the AutoAlbument setups had the best validation accuracies and therefore were not chosen for the final model, this has not been further evaluated.

Domain gap and generalization

In this section, we examine whether a domain gap between the Nikiforov dataset and the Tharun and Thompson dataset exists. This is done to improve the interpretability of the generalization ability experiments carried out afterwards. The generalization ability of the classification methods is analyzed by training the methods on one dataset and applying them to the other one.

Domain gap

Although the images in both datasets are hematoxylin and eosin stained and show thyroid gland tumors, there is still a domain gap, i.e., due to the used microscope and imaging settings. Furthermore, the tumor subtypes in the datasets can differ. The images in the Nikiforov dataset have a resolution of 0.49 µm/px and the images in the Tharun and Thompson dataset have a resolution of 0.23 µm/px. For the feature-based classification, the Nikiforov dataset has been upscaled by the factor of two. This has been done to use the same segmentation network for the Nikiforov dataset and the Tharun and Thompson dataset. Upscaling by the factor of two leads to a resolution of 0.245 µm/px which still differs by 6.5% compared to the Tharun and Thompson dataset.

To examine whether a domain gap is present, we used a ResNet50 pretrained on ImageNet and applied it to the images of both datasets. The 2048 output features of the second to last layer are used to describe each image. To visualize the features, the dimensionality is reduced from 2048 features to two features per image by the unsupervised dimension reduction approach UMAP [3].

To match the resolutions between both datasets, the experiment is performed once with the Tharun and Thompson dataset downscaled by the factor of two and once with the Nikiforov dataset upscaled by the factor of two. The result is shown in Figure S3. Although, the ResNet50 was not trained on histopathological data, UMAP was able to clearly separate both datasets.

Both datasets are mostly represented by compact, separate distributions in the reduced feature space. Some outliers exist in Figure S3a for the Tharun and Thompson dataset. In Figure S3b, some samples

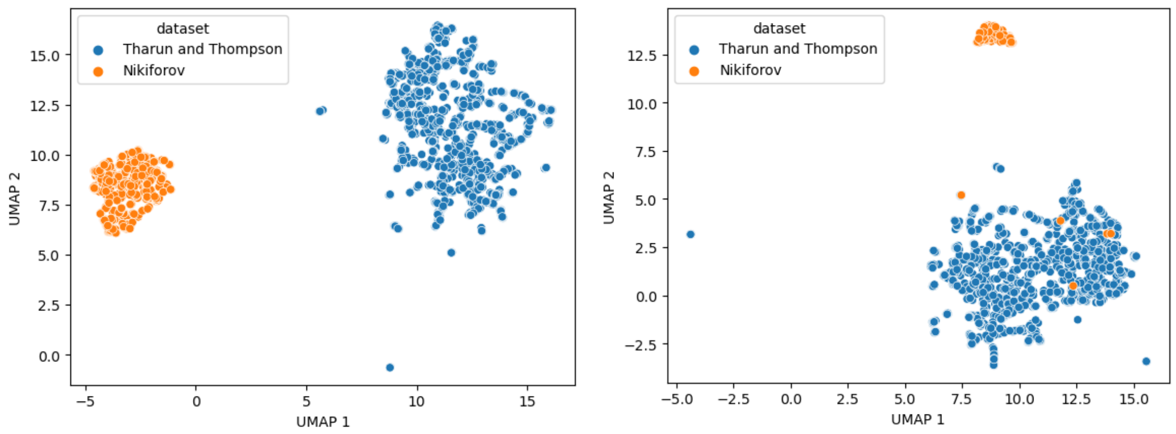

(a) Native Tharun and Thompson dataset resolution **(b)** Native Nikiforov dataset resolution

Fig S3. Visualization of the domain gap between the Nikiforov dataset and the Tharun and Thompson dataset. UMAP is used to reduce the 2048 neural network features to two (UMAP 1 and UMAP 2). In (a) the Nikiforov dataset is upscaled by the factor of two, while in (b) the Tharun and Thompson datset is downscaled by the factor of two.

of the Nikiforov dataset are in the Tharun and Thompson dataset distribution. Visual inspection of this samples yielded no abnormalities compared to the remaining dataset. Therefore, it can be concluded that a domain gap is present for both datasets.

Generalization ability

To evaluate the accuracy of the trained classification approaches on new datasets, the Tharun and Thompson dataset is used for training while the Nikiforov dataset is used for evaluation and vice versa.

Feature-based classification For the evaluation of the generalization ability, we use the same trained classifiers performing best for the evaluation on a single dataset. For the Tharun and Thompson dataset this is a SVC with quantile transformation, no standard scaling and sequential feature selection with a total of 22 features selected. For the Nikiforov dataset, this is a SVC with quantile transformation, standard scaling and χ^2 feature selection with a total of 22 features selected. Because the classifier hyperparameters are optimized with nested cross-validation with five splits, the final accuracy is the average of the performance of five different trained models. For the evaluation of the generalization ability, we use the same five trained models used for the accuracy on one dataset and apply them to all samples of the other dataset. The final prediction is defined by the majority vote of the five models. The results are shown in Table S4a.

Using the models trained on the Tharun and Thompson dataset and applying them to the Nikiforov dataset results in an accuracy of 66.2%. This is 17.3% lower than the best feature-based classification trained and evaluated directly on the Nikiforov dataset.

Using the models trained on the Nikiforov dataset and applying them to the Tharun and Thompson dataset results in an accuracy of 71.2% which is 18.5% lower than the best feature-based classification trained and evaluated directly on the Tharun and Thompson dataset.

Since the pixel resolution of both datasets differs by 6.5% after scaling and shape features like the area are among the most important features, this could be a reason for the decrease in accuracy. In the future, this could possibly be reduced by scaling the final extracted features regarding the training dataset resolution. Furthermore, e.g. image scaling, microscope properties, illumination and sample preprocessing can affect the color features. Change in color can subsequently change the cell segmentation

Table S4. Accuracy for the classification methods trained and evaluated on the same or a different dataset. The Tharun and Thompson dataset is abbreviated by T&T.

(a) Feature-based classification

	test data	
training data	Nikiforov	T&T
Nikiforov	83.5%	71.2%
T&T	66.2%	89.7%

(b) Deep learning-based classification

	test data	
training data	Nikiforov	T&T
Nikiforov	77.4%	70.5%
T&T	62.4%	89.1%

and therefore the shape and spatial features. Reducing this impact is not trivial and we aim to investigate this in the future.

Deep learning-based classification Similar to the feature-based classification, the accuracy on a single dataset is the average of five different trained models. This is due to the fact, that a cross-validation with five splits is used for the deep learning-based classification. Again, all five models trained on the different splits of one dataset are applied to the other dataset and the final prediction is defined by the majority vote of the five models. Since the models are trained on different resolutions, the test dataset is scaled according to the training dataset. The results are shown in Table S4b.

The models trained on the Tharun and Thompson dataset and applied to the upscaled Nikiforov dataset result in an accuracy of 62.4% which is 15.0% lower than the accuracy of the deep learning-based classification models trained directly on the not scaled Nikiforov dataset.

The models trained on the Nikiforov dataset and applied to the downscaled Tharun and Thompson dataset result in an accuracy of 70.5% which is 18.6% lower than the accuracy of the deep learning-based classification models trained directly on the not scaled Tharun and Thompson dataset.

A possibility to increase the generalization ability of the deep learning-based classification could be test time augmentation [4]. Furthermore, we think that especially the deep learning-based classification will highly benefit from a more diverse training dataset covering a larger feature space enabling it to generalize better on new data.

References

1. Haralick RM, Shanmugam K, Dinstein I. Textural features for image classification. IEEE Trans Syst Man Cybern. 1973;SMC-3(6):610–621. doi:10.1109/TSMC.1973.4309314.

2. Shannon CE. A mathematical theory of communication. The Bell System Technical Journal. 1948;27(3):379–423.

3. McInnes L, Healy J, Melville J. UMAP: Uniform manifold approximation and projection for dimension reduction. 2018. Available from: `https://arxiv.org/abs/1802.03426`.

4. Wang G, Li W, Ourselin S, Vercauteren T. Automatic brain tumor segmentation using convolutional neural networks with test-time augmentation. In: Crimi A, Bakas S, Kuijf H, Keyvan F, Reyes M, van Walsum T, editors. Brainlesion: Glioma, Multiple Sclerosis, Stroke and Traumatic Brain Injuries. Cham: Springer International Publishing; 2019. p. 61–72.
