# Supplementary figures and images for: Machine learning methods for automated classification of tumors with papillary thyroid carcinoma-like nuclei: A quantitative analysis
Source: PLoS One. 2021 Sep 22;16(9):e0257635. doi: 10.1371/journal.pone.0257635 (PMC8457451; doi:10.1371/journal.pone.0257635)

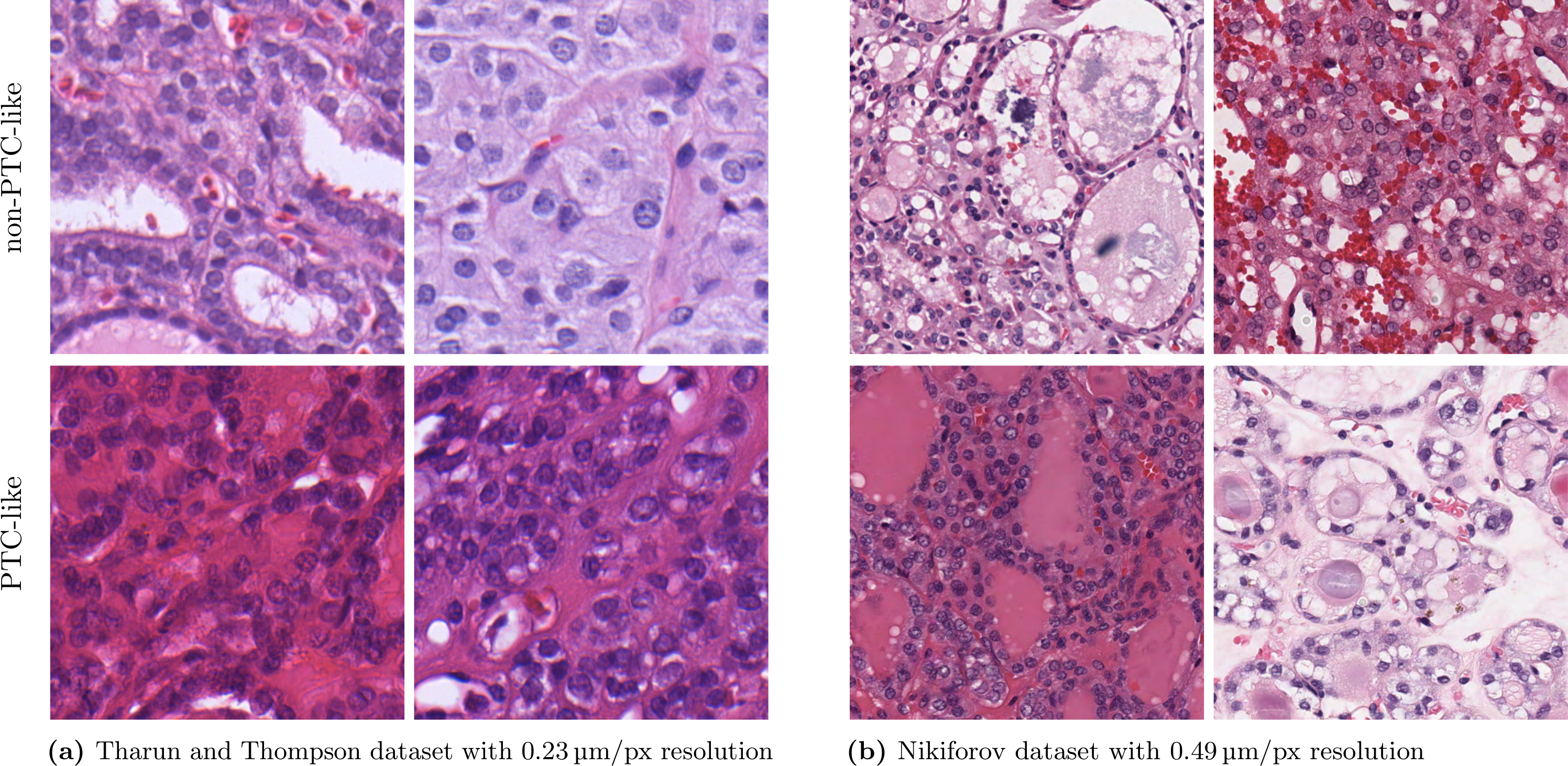

Supplement: S1 Fig — Example images from the Nikiforov dataset and the Tharun and Thompson dataset from both classes PTC-like and non-PTC-like. (PNG) [file pone.0257635.s002.png]
